# The school environment-related predictors of fights and bullying among School-Age Children in Serbia

**DOI:** 10.1093/eurpub/ckac129.521

**Published:** 2022-10-25

**Authors:** M Šantrić Milićević, S Stankovic, D Nikolić, N Bjelica, U Babić, Lj Rakić, J Todorović, Z Terzić-Šupić

**Affiliations:** 1 School of Public Health and Health Management, Faculty of Medicine University of Belgrade, Belgrade, Serbia; 2 Institute of Social Medicine, University of Belgrade Faculty of Medicine, Belgrade, Serbia; 3 Faculty of Medical Sciences, University of Kragujevac, Kragujevac, Serbia; 4 Physical Medicine and Rehabilitation, University Children’s Hospital, Belgrade, Serbia; 5 Directorate, Primary Health Care Centre “Dr Simo Milosević”, Belgrade, Serbia; 6 University Clinical Centre of Serbia, Faculty of Medicine, University of Belgrade, Belgrade, Serbia; 7 Clinic for Hematology, University Clinical Centre of Serbia, Belgrade, Serbia

## Abstract

**Background:**

The health and development of school-age children is a contemporary topic of various health policies and programs, which has become even more of a focus in critical situations such as the COVID-19 pandemic. The study aims to assess the prevalence of school-age children's participation in fights and bullying in Serbia, and to examine the relevance of students’ socio-demographic characteristics and perceptions of school and relations with other students and professors for participation in fights and bullying.

**Methods:**

A secondary analysis of the original data of the 2017 HBSC study is performed on 3267 students in a nationally representative sample of primary and high schools in Serbia. Predictors of taking part in fights and taking part in bullying were examined by using univariate and multivariate logistic regression.

**Results:**

The main results show that 50.8% of boys and 17.1% of girls have taken part in fights, while 17.7% boys and 10.4% of girls have taken part in bullying. Students who felt a large and very large burden of school obligations were 1.43 times more likely to participate in bullying at least once, while they were 1.38 and 2.12 times more likely to participate in multiple fights and 4.04, 1.24, and 2.78 times more likely to participate multiple times in bullying. Fights among school-age children are significantly positively associated with living with relatives/legal guardians and poor quality of life.

**Conclusions:**

The prevalence of participating in at least one fight/bullying is higher than in multiple fights/bullying. These associations suggest a necessity to enhance the monitoring and control of peer behavior among school-age children. The findings of the study imply key enablers of protection, such as building relationships based on team spirit and work, friendly behavior, empathy, and help, which should be included in the value system of school and family activities in programs to combat fights and bullying in school-age children.

**Key messages:**

• In Serbia, every second boy and every fifth girl participated in fights, while less than every fifth boy and every tenth girl participated in bullying.

• Study results can inform school and healthcare actors’ efforts to improve school-age children’s development and health capacity for life.

